# Artificial Intelligence: An Interprofessional Perspective on Implications for Geriatric Mental Health Research and Care

**DOI:** 10.3389/fpsyt.2021.734909

**Published:** 2021-11-15

**Authors:** Brenna N. Renn, Matthew Schurr, Oleg Zaslavsky, Abhishek Pratap

**Affiliations:** ^1^Department of Psychology, University of Nevada, Las Vegas, NV, United States; ^2^Department of Biobehavioral Nursing and Health Informatics, University of Washington, Seattle, WA, United States; ^3^Krembil Centre for Neuroinformatics, Centre for Addiction and Mental Health, Toronto, ON, Canada; ^4^Vector Institute for Artificial Intelligence, Toronto, ON, Canada; ^5^Department of Biomedical Informatics and Medical Education, University of Washington, Seattle, WA, United States; ^6^Institute of Psychiatry, Psychology & Neuroscience, King's College London, London, United Kingdom

**Keywords:** machine learning, deep learning, psychotherapy, older adults, technology, depression, natural language processing, personalized medicine/personalized health care

## Abstract

Artificial intelligence (AI) in healthcare aims to learn patterns in large multimodal datasets within and across individuals. These patterns may either improve understanding of current clinical status or predict a future outcome. AI holds the potential to revolutionize geriatric mental health care and research by supporting diagnosis, treatment, and clinical decision-making. However, much of this momentum is driven by data and computer scientists and engineers and runs the risk of being disconnected from pragmatic issues in clinical practice. This interprofessional perspective bridges the experiences of clinical scientists and data science. We provide a brief overview of AI with the main focus on possible applications and challenges of using AI-based approaches for research and clinical care in geriatric mental health. We suggest future AI applications in geriatric mental health consider pragmatic considerations of clinical practice, methodological differences between data and clinical science, and address issues of ethics, privacy, and trust.

## Introduction

Artificial intelligence (AI) learns patterns in large multimodal datasets both within and across individuals (1) to help improve understanding of current clinical status [e.g., calculating a risk score for heart disease (2)] or predict a future outcome [e.g., predicting daily mood fluctuations (3)]. Such technology is increasingly critical and opportune in our digital healthcare revolution. Advances in technology, such as the ubiquity of smartphones, other wearables, and embedded sensors, in addition to the emergence of large datasets (e.g., electronic health records) have altered the landscape of clinical care and research. AI approaches can dynamically interpret such complex data and generate incredible insight to potentially improve clinical methods and results. AI holds the potential to revolutionize geriatric mental health care and research by learning and applying such individualized predictions to guide clinical decision-making. Specifically, AI can contribute to the proactive and objective assessment of mental health symptoms to aid in diagnosis and treatment delivery to suit individual needs, including long-term monitoring and care management.

The big promise for AI in mental health care and research—largely due to its reliance on big data—is to facilitate understanding of what works for whom, and when. However, much of this momentum is driven by machine learning experts (e.g., data and computer scientists and engineers) and runs the risk of being disconnected from pragmatic issues in clinical practice. In this piece, we bring the perspectives of clinician-scientists in clinical geropsychology (BNR and MS) and geriatric nursing (OZ) to bear on expertise in AI and data science (AP). We provide a brief overview of AI in mental health with the main focus on possible applications and challenges of using AI-based approaches for research and clinical care in geriatric mental health.

## Clinical Applications of AI

**Figure 1:**
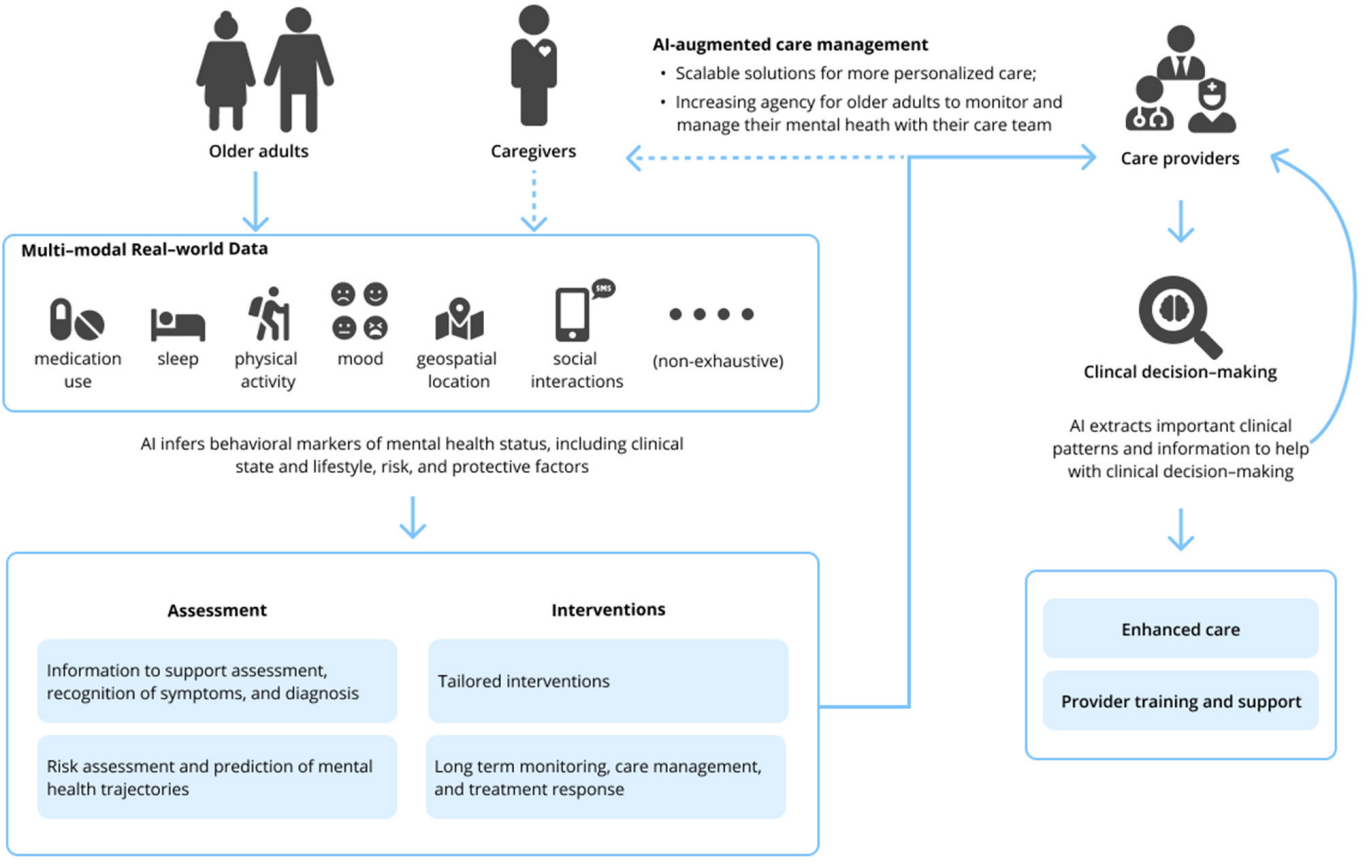
Clinical opportunities for artificial intelligence in geriatric mental health care.

The field of geriatric mental health focuses on both normal and pathological aging from a biological and psychological perspective; this encompasses acute and chronic physical illness, neurodegeneration and cognitive impairment, and mental disorders in people aged 65 and older. Research and clinical applications within geriatric mental health focus on both care delivery and the evaluation, diagnosis, prevention, and treatment of such disorders. The appeal of such AI-enabled technology to advance geriatric mental health care is 2-fold. First, AI technologies hold the potential to develop precision models that are both personalized and conceivably more accurate than traditional clinical care using vast amounts of real-world multimodal data about patients, including the influence of environmental and other risk and protective factors. Secondly, technology in general has long been heralded as a means to overcome traditional access barriers of cost, time, distance, and stigma, all of which are relevant for older adults. While a thorough review of AI is beyond the scope of this Perspective, relevant machine learning (ML) and deep learning applications [including natural language processing (NLP)] of AI are presented in Table 1. Interested readers are directed to other reviews for more in-depth descriptions of AI in mental health (10–13). We briefly review three clinical domains relevant to geriatric mental health care below and subsequently suggest specific areas where AI can assist clinical care (see Figure 1).

**Table 1:** Overview of artificial intelligence (AI) technologies with relevance to geriatric mental health.

**Type of AI technology**	**Definition**	**Clinical example**
Machine learning (ML)	A family of statistical techniques that allow computer programs to make predictions and decisions based on past data.	
Supervised	A type of machine learning that uses labeled datasets to “train” algorithms. For example, a dataset includes a label for cognitive impairment (cognitively impaired or not). The model learns on a set of training data, then the algorithm is tested on unlabeled data to ensure its accuracy in classifying the target variable.	Modeling a variety of clinical, lifestyle, and sociodemographic factors to help predict cognitive function in older people; clinicians could use this non-invasive screening method to decide whether or not a patient warrants further in-depth cognitive assessment (4).
Unsupervised	A type of machine learning based on analyzing unlabeled data to discover hidden patterns or data groups. The algorithm is not provided with a label thus, subject-matter experts must evaluate the data output to ensure its usefulness. Unlabeled data are sorted into groups or patterns to identify the underlying structure of the data.	Identifying high likelihood of dementia in population-based surveys (5).
Deep learning (DL)	A subfield of machine learning; deep learning models use computer programs called artificial neural networks to discover latent relationships in complex, raw data. DL algorithms develop multiple hierarchical layers of data representation and learn complex underlying patterns.	A trial in India used deep learning to predict depression among older adults and had a high prediction accuracy (97.2%) based on sociodemographic variables and morbidity (sleep difficulties, mobility difficulty, hearing, and visual impairment) (6).
Natural language processing (NLP)	Natural language processing (NLP) aims to comprehend human language by extracting word features (such as syntax, grammar, and semantic meaning) from text and transcribed speech. It holds much potential in mental health research and care, where text (e.g., electronic health records [EHRs]) and speech (e.g., psychotherapy session content) are key real-world data sources.	Using speech features (e.g., speech fluency, prosody, duration) to detect late-life depression (7).
Computer vision	Computer vision is used to detect and classify objects. The model imposes a grid-like structure on images and learns key features, such as edges and curves, to build a unique model to recognize similar objects.	Extracting gait features from video recordings of older adults with dementia (“human pose estimation”) to predict future falls (8).
Reinforcement learning	Deep reinforcement learning (RL) is a form of adaptive learning that rewards desired outcomes (behaviors) and penalizes undesirable or unwanted outcomes. Such algorithms learn to sense and interpret the right and wrong actions in an environment and train through trial and error.	Helping providers by editing written therapeutic exchanges to increase the level of expressed empathy, a critical component of therapeutic conversations (9).

### Assessment, Symptom Recognition, and Diagnosis

A major issue in geriatric mental health care and research is accurate classification of a disorder. Many mental health conditions, including late-life depression, go undetected and untreated (14). When symptoms are recognized, diagnosis primarily relies on subjective recollections of symptoms, which leads to a considerable amount of diagnostic variability and may be subject to patient recall bias (15). Moreover, differential diagnosis can be particularly challenging in older adult patients with multimorbidities or when considering conditions with overlapping symptoms. A compelling application of AI is accurately predicting who needs mental health treatment before someone realizes they need it—or, before symptoms become too burdensome—by tracking early cues related to a change in an individual's daily behavior. One of the most ubiquitous opportunities is personal sensing, which converts the huge amount of sensor data collected by our phones (or other wearable devices) into clinically meaningful information about behaviors, thoughts, and emotions to make inferences about clinical status and/or disorders (16). These data sources can be rich and multimodal, encapsulating sleep, social interaction, and physical activity, to name a few features. Such data may serve as objective measures for hallmark symptoms (e.g., fatigue and sleep disturbances) in the diagnosis of depression in older adults (17).

A small but growing body of literature has begun to apply AI approaches to geriatric mental health assessment and diagnosis, largely in the context of depression (10) and neurocognitive impairment (11). For example, language ability and processing—including spontaneous speech—is often an early affected cognitive domain in the course of dementia, especially Alzheimer's disease, and has been proposed as a target for early recognition and diagnosis (18). However, traditional methods of early recognition and diagnosis often produce significant overlap with “normal” cognitive functioning among older adults, and thus have reduced clinical utility in early detection (19). AI techniques such as NLP may detect speech features (e.g., acoustic features such as pause duration and emotion) that are sensitive to cognitive decline and may better differentiate those with early impairment than traditional neuropsychological assessment (19, 20).

### Treatment and Treatment Monitoring

The shortage of geriatric mental health specialists (21) and barriers to treatment seeking among older adults (22–24) mean that patients with mental health needs are often delayed in obtaining treatment, if they receive treatment at all. As it stands in current clinical practice, access to evidence-based treatment is often limited (25), and when implemented, treatment decisions are often guided by trial and error. Ongoing assessment is also crucial to assess effectiveness of treatment, but may be overlooked or untenable in routine practice, rendering ineffective treatment decisions. As AI aims to predict who needs mental health services, the next compelling application of such technology will be to answer the question of “What works for whom, and when?” A promising application of AI for mental health, inspired by precision medicine, is to identify subgroups of patients with similar symptom expressions and outcomes to guide treatment decisions, commonly referred to as “subtyping” (26). Once treatment is initiated, AI could also help clinicians monitor response to treatment and symptom trajectory, such as through passive recording of behavioral data using wearable sensors.

Another example to optimize treatment is to quickly mobilize tailored supports using just-in-time adaptive interventions. These adapt the type, timing, and intensity of treatment based on the individual's need at the moment and context they most need the support (27). Such efforts in geriatrics include computational modeling based on smartphone data to target health behavior change (i.e., low physical activity and sedentary behavior) in older adults (28). These models use sensor data to monitor health states with the goal of delivering personalized interventions to mitigate behavioral and psychological factors that contribute to health risk.

Intelligent voice assistants, virtual health agents, and conversational agents (e.g., chatbots) are designed to reduce health care system burden (29) and improve patient autonomy and self-management (30). While mainstream conversational agents have yet to be tested with older adults, preliminary evidence suggests that older adults are comfortable self-disclosing with other conversational agents (19). It is conceivable that such AI may one day be used to support “aging in place,” such as allowing older adults to complete remote assessments for routine monitoring. Researchers are also prototyping AI-based “smart homes” to support safety and independence among older adults and individuals with disabilities and chronic conditions (31). However, ongoing engagement is required for AI to assist with long-term monitoring or treatment delivery. For example, while intelligent voice assistants such as Amazon's Echo have the potential to support independence among older adults, users may discontinue such products if they do not realize benefits or experience challenges using such devices in shared spaces (32).

### Clinical Decision-Making, Provider Training and Support

AI may free up time for the clinician to implement treatment decisions and focus on other therapeutic targets (e.g., client rapport) where the application of current AI technologies has been ineffective (33). AI-based data collection and harmonization may streamline patient flow, automate assessments, monitor longitudinal trajectories and outcomes, reduce paperwork, and monitor medication(s) and potential contraindications (34), thus freeing providers to practice the “human” elements of mental health care. AI may also be used to train mental health professionals. This is particularly relevant to the current geriatric workforce shortage (21). Examples include virtual patient simulations to train and evaluate clinical skills (e.g., asking proper diagnostic questions) (35) and NLP to analyze the quality of engagement between a therapist and a patient in a psychotherapy session (36). However, limitations to this technology remain; this work found models only modestly predicted patient-rated alliance from psychotherapy session content (36).

## Challenges and Opportunities

Now we want to highlight some challenges and propose how AI solutions can be applied to real-world problems in geriatric mental health care and research. We suggest the AI community partners with clinician-researchers and care teams (including nursing staff, providers, and caregivers), and vice versa, in order to make most relevant the potential of such technology. This is particularly germane to issues of geriatric mental health care.

### Unique Challenges in Geriatrics

Aging is a complex process that involves interconnected changes spanning cellular to psychological to sociocultural processes, the results of which present unique challenges when working in geriatrics. First, older adults are less likely than younger adults to receive accurate diagnosis and treatment for mental health issues (37), and barriers are greater among racially and ethnically diverse older adults compared to their non-Latino White counterparts (23). Workforce shortages, specifically lack of providers with competencies in the specific needs of older adults, contribute to these issues (21). Older adults also present with greater comorbidity, chronicity, and complexity than their younger adult counterparts; acute and chronic physical health conditions, medication use, and cognitive, sensory, or functional impairments can all complicate the detection and diagnosis of a mental health condition. Additionally, the variation in manifestation of mental health symptoms and treatment responses in older adults affects timely and accurate diagnosis. For example, an enduring finding in geriatric mental health care is that older adults with depressive symptoms are less likely than younger adults to present with sadness and are more apt to endorse anhedonia (loss of interest or pleasure), apathy, and somatic symptoms such as fatigue, diffuse aches and pain, or malaise (38). Somatic symptoms of late-life depression also overlap with symptoms of chronic disease, potentially obscuring or complicating diagnosis of mental health conditions. Moreover, older adults may be poor utilizers of mental health services if they are uncertain whether their symptoms are due to psychological problems or normal aging (39). Thus, AI holds promise to capture real-world behavioral data to aid in the recognition and diagnosis of mental health conditions in older adults. The majority of the literature points to applications of AI among younger adults (often college-aged convenience samples). Next steps are to prototype, train, and validate AI approaches on data from diverse respondents, including older adults, to capture the specific clinical needs and heterogeneity in the population.

Social, environmental, and familial contexts are important considerations in geriatric mental health. Caregiving is one such relevant factor. Persons with chronic or life-limiting disease–often older adults—require progressively extensive attention and assistance with activities of daily living. This care is often provided by family members or other unpaid caregivers. AI technologies may better prepare and support caregivers in their tasks. A systematic review of 30 studies (40) described a range of assistive AI devices designed to facilitate caregiving, such as support with dressing or handwashing or detecting falls. However, the review noted that most studies were descriptive or exploratory, offering very limited evidence of such technology to date.

Social factors such as social isolation and loneliness may also exacerbate mental health issues; indeed, a recent federal report highlighted the epidemic of social isolation and loneliness among older adults (41). AI could be used to both assess and offer supports for loneliness. For example, a proof-of-concept study used NLP to identify loneliness among U.S. community-dwelling older adults based on speech from qualitative interviews (42). Importantly, this study attempted to understand sex differences in the reporting of such a complex psychological construct—something with which clinicians may struggle. As with much of the AI applications to date in geriatric mental health, the authors note that future work will need larger, more diverse samples and to incorporate multimodal data streams to improve the predictions. In any case, AI supports designed for older adults will need to address not only psychological and biological/medical factors, but social and environmental factors to be most relevant.

The term “older adult” encompasses a wide range of the lifespan and includes diverse individuals from various birth cohorts; racial/ethnic, cultural and socioeconomic backgrounds; and functional abilities. As healthcare in general, and AI opportunities specifically, relies on technology, there is concern that older adults will be left out of such a digital health revolution. Even though many members of the “young-old” (65–74 years) and older cohorts may be accustomed to smart devices and other technologies, older adults are often left out of the design and marketing of such innovations (43). Sensory issues, ranging from tremors to limited vision, may also impede the use of conventional technological devices designed for users with normative abilities. When innovations are marketed toward older people, they often reflect a pathological view of aging and are limited to support for emergency monitoring (e.g., fall detection). Our call to action is that AI developers leverage a user-centered perspective, including diverse older adults with a range of health-related quality of life, during the design and evaluation (44) to uncover such technology's viability and fit-for-purpose in the target population.

### Methodological, Practical, and Other Challenges

Given the pursuit of such rapid and novel innovation, not all AI developments will readily translate to clinical or other real-world settings. While not exhaustive, we outline a few key challenges in an attempt to bridge data science with clinical science in geriatric mental health and suggest next steps in addressing such challenges.

First, there has been a paradigm shift away from traditional experimental studies that typify mental health research to rapid innovations in AI (13). The empirical approaches familiar to clinicians—namely hypothesis testing and reliance on evidence-based practice—are potentially at odds with the proof-of-concept, hypothesis-generating demonstrations that characterize much AI research to date. The innovations propelling AI forward are often tested on small samples to demonstrate proof-of-concept (40); however, this runs the risk that ML models will be overfit, leading to spurious findings and lacking generalizability to new data sources. External validation of the model (that is, testing in new datasets) is essential to improve prediction, yet only three of 51 studies in a recent review of ML in psychotherapy research did so (45). When large datasets are available, they are often prone to bias arising from differential recruitment, attrition, and engagement over time (46). Importantly, adults over the age of 60 are those least represented in digital health study samples, and such studies rarely reflect the racial/ethnic and geographical diversity of the U.S., limiting the validity of findings (46). Moreover, researchers from non-health science fields may use different reporting norms than clinician scientists, resulting in missing key pieces of information, including participant demographics and other aspects of methods (e.g., location of data collection) (40), which limit inferences and generalizability.

When it comes to implementation of AI, clinicians may override algorithm-based recommendations, or patients may be wary of algorithm-recommended treatment. Although computational modeling is a powerful tool to sift through predictors to develop complex algorithms, the “black box” of such computations may be off-putting to clinicians who have long relied on their own clinical reasoning to drive decision-making, or who may not fully understand the statistical models (47). Moreover, algorithm recommendations may not fully incorporate all clinical considerations, including patient restrictions or preferences. A major pitfall of using AI for mental health care—geriatric or otherwise—is that such systems will sometimes be wrong, resulting in patient harm. For example, a patient with a depressive disorder may be misclassified and not treated. While such error happens in human-based decision making, it will be important to build in safeguards when implementing such AI systems at scale (e.g., transparency around computational inferences and classification; routine clinician assessment to augment such AI classification for greater reliability; development of other safety nets in healthcare). Finally, even if we could use AI to accurately predict clinical state or worsening of a patient *via* sensor data or other algorithmic prediction, what would a clinic or individual clinicians actually do with such data? A clear bridge between developing and implementing such predication-based models is developing appropriate clinical workflows and interventions to address such predictions.

Data scientists must also partner with clinicians and clinical scientists to ensure that data features are meaningful and valid for older adults (16). In our own work using ML to model daily variation in depressive symptoms based on mobility data, we were unable to access raw mobility data from the proprietary sensor software and translate such data into meaningful variables (48). We also ran into issues with intra- and interindividual variation in phone usage patterns—data are only as robust as the degree to which users use the device (3, 48), which may vary between older and younger adults. More work is needed to understand older adults as unique users of devices, such as smartphones, rather than simply extrapolating assumptions from younger users. Finally, sensors and other multimodal sources of data may detect incredible variability in clinical states and behavioral markers. However, for practical utility, AI models need to be trained to differentiate features that are clinically relevant—that is, diagnostic—from transient mood states. This will again require models based on large and diverse samples of older adults to ascertain features associated with geriatric mental health conditions.

One cannot tread into the topic of AI without running into discussion of ethics, structural inequalities, privacy, and trust issues. A full discussion of these topics is beyond the scope of this paper but has been discussed elsewhere (49–51). Briefly, these will be critical issues to consider as the innovation of data science meets the practical applications of clinical work. For example, what are the bioethical considerations if an AI algorithm recommends a particular intervention, which the clinician decides against it, and the patient decompensates? Or, conversely, where does liability lie if a patient dies after a clinician deploys an algorithm-recommended treatment (52)? It is also crucial to acknowledge that racial, gender, and ageist biases and discrimination are deeply embedded in healthcare—and as a result, in the AI systems that learn from such data sources. When unchecked, the inferences drawn from such technologies are likely to perpetuate systemic injustices in healthcare. These may result from bias and a lack of transparency in developing algorithms, such as using training data from a preponderance of young White men or using flawed proxy variables to calculate risk scores (53). Such bias is then further maintained in how providers respond to such algorithmic predictions. Thus, understanding and preventing the root causes for bias in AI systems must be a priority to monitor and mitigate such consequences. Privacy concerns among users of various technology-based assessments and interventions has also been a central theme arising in research from our group (54–56). Trust may vary as a function of who is conducting the research—for example, trust in internet-based research is higher (and participants more likely to share their data) when the research is conducted by university researchers compared to private companies (55). Building trustworthiness of AI in geriatric mental health care and research will rely on reconciling some of the issues discussed above—namely, *explainability* (the ability to understand or describe how a model arrived at its prediction), *transparency* (clear and transparent methodology), and *generalizability* (related to methodology; exhaustive testing and validation of models) (57).

## Conclusion

AI holds promise for more accurate diagnosis and personalized treatment recommendations, yet the field is nascent with no established pathway for integration into routine clinical care. A recent market research survey found that healthcare providers remain highly skeptical of consumer technology, remote data collection, and the integrity of such data (58). Moreover, development and implementation of such technology must incorporate clinicians, patients, and caregivers as key stakeholder groups to build trust and adopt user-centered approaches that address privacy and usability issues. We may be on the cusp of a new era that will allow the full potential of AI to take hold in mental health care broadly, and geriatrics specifically. However, until clinicians join forces with data scientists, engineers, and developers—and until such technology addresses the pragmatic concerns that clinicians and patients face—we will only scratch the surface of such potential for these technologies.

## Author Contributions

BNR and AP conceptualized and designed the work. BNR, MS, and AP drafted the manuscript. All authors critically read and revised the manuscript for important intellectual content and approved the submitted version.

## Funding

This project was supported in part by the National Institute of Mental Health [grant P50 MH115837] and the National Institute on Aging [grant K23 AG059912]. AP's effort was supported by a grant from the Krembil Foundation, Toronto, Canada. Open access publication fees were provided by BNR's startup funds from the University of Nevada, Las Vegas. The sponsors played no role in the design of this manuscript, nor did they have any role during its execution or decision to submit.

## Conflict of Interest

BR receives unrelated research support from Sanvello. The remaining authors declare that the research was conducted in the absence of any commercial or financial relationships that could be construed as a potential conflict of interest.

## Publisher's Note

All claims expressed in this article are solely those of the authors and do not necessarily represent those of their affiliated organizations, or those of the publisher, the editors and the reviewers. Any product that may be evaluated in this article, or claim that may be made by its manufacturer, is not guaranteed or endorsed by the publisher.
